# Protein‐Based High‐Performance Liquid Chromatography and Cyclodextrin‐Capillary Electrokinetic Chromatography for the Chiral Separation of Azoles

**DOI:** 10.1002/elps.70124

**Published:** 2026-06-23

**Authors:** Sarah Moreau, Amy Dillon, Giacomo Russo, Susanne K. Wiedmer

**Affiliations:** ^1^ Department of Chemistry University of Helsinki Helsinki Finland; ^2^ Centre for Biomedicine and Global Health School of Applied Sciences Edinburgh Napier University, Sighthill Campus Edinburgh UK

**Keywords:** azoles, capillary electrokinetic chromatography, chiral separation, cyclodextrins, dual cyclodextrin system, enantioseparation, human serum albumin

## Abstract

Liquid chromatographic (LC) and capillary electrokinetic chromatography (EKC) methods with ultraviolet/diode array detection were developed for the enantioseparation of five azoles: econazole (ECO), miconazole (MICO), imazalil (IMA), ketoconazole (KET), and penconazole (PEN). The chiral selectors investigated were human serum albumin (HSA) and eight cyclodextrins (CDs), exploited in LC and EKC, respectively. HSA LC offered partial enantioseparation of IMA and, less effectively, of PEN. Interestingly, these azoles share a high degree of molecular similarity, as confirmed by in silico calculations. This may suggest that a single enantiomer achieves preferential access to enantioselective sites of the protein. To address the limited enantioselectivity of HSA for these azoles, CDs were explored as chiral selectors where EKC was preferable to LC due to its superior separation efficiency. The chiral discrimination capabilities of eight CDs were evaluated for these azole derivatives, aiming for fast separations with minimal amounts of CDs. Effective enantioseparation was achieved for ECO, MICO, IMA, and KET, using two of the tested CDs: 2‐hydroxypropyl‐β‐cyclodextrin (HP‐β‐CD) and heptakis(2,3,6‐tri‐*O*‐methyl)‐β‐cyclodextrin (TM‐β‐CD). Satisfactory resolutions were obtained using a 40 mM phosphate buffer (pH 3.2) with 1 mM HP‐β‐CD for ECO and MICO and 8 mM TM‐β‐CD for KET. For IMA, the optimal conditions consisted of a 20 mM phosphate buffer with 3 mM HP‐β‐CD. None of the tested CDs were effective for the enantiomeric separation of PEN; therefore, a dual CD system was developed for the first time for this compound, employing succinyl‐β‐CD (Succ‐β‐CD) in combination with HP‐β‐CD in a borax buffer under basic conditions (pH 9.4), enabling complete enantioseparation of PEN. The low concentration of CDs required under optimal conditions renders the developed methods highly cost‐effective.

## Graphical abstract

Human serum albumin‐based high‐performance liquid chromatography and cyclodextrin‐based capillary electrokinetic chromatography were used for the chiral separation of azoles. Although only two of the five tested azoles could be enantioseparated by liquid chromatography, all of them were separated by capillary electrokinetic chromatography using single or dual cyclodextrin solutions.

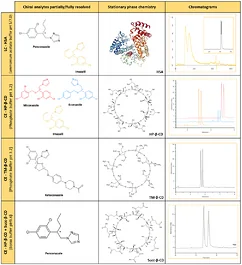

## 1 Introduction

Chirality plays a significant role in modern pharmacotherapy, whereby approximately 56% of drugs administered to patients are chiral molecules with nearly 88% marketed as racemic mixtures. Regrettably, the use of racemates is largely inefficient as it involves the unnecessary consumption of resources, that is, the distomer of the chiral medicine, often resulting in an increased risk of adverse effects. This is due to the aspect that in many cases, a single enantiomer of a chiral molecule can exhibit higher biological activity at the target binding site than its opposing absolute configuration [1]. Reflecting this significance, chirality remains prominent in drug development. Notably in 2020, 20 of the 35 pharmaceuticals approved by the US Food and Drug Administration (FDA) were chiral, and a sustained increase in regulatory submissions for chiral drugs has been observed [2].

In parallel, medicinal chemistry increasingly focuses on the development of enantiomerically pure compounds to enhance efficacy while mitigating toxicity and adverse effects associated with the distomer. Nevertheless, these pharmacologically ineffectual enantiomers remain environmentally relevant, as they may exhibit significant toxicity and persistence once released into the environment. Their growing presence has contributed to the emergence of a new class of pollutants, commonly referred to as “emerging contaminants.” Such emerging contaminants pose substantial challenges in biomedicine and ecology due to their biological activity even at trace concentrations and their potential impact on both ecosystems and human health [3].

Given this context, chiral separation techniques are critical for pharmaceutical development, environmental monitoring, and regulatory compliance. With the global market for chiral technologies projected to reach approximately $150 billion by 2030, this is a pivotal moment underscoring the need for robust, selective, and sustainable analytical approaches [2] to be applied. Moreover, the general policies of the US FDA, the European Medicines Agency (EMA), and the UK's Medicines and Healthcare Products Regulatory Agency (MHRA) require preclinical and clinical characterization of each component of a stereoisomeric mixture for market approval [4, 5]. Consequently, the ability to separate and analyze these enantiomers is paramount for patient safety, drug development, quality control, and regulatory compliance [6].

Among the various methods employed for chiral separation, capillary electromigration techniques (CE) and liquid chromatography (LC) have emerged as powerful techniques due to their rapid analysis times, minimal sample requirements, and high resolution [7, 8]. Chiral LC is a highly versatile technique, owing to the wide availability of chiral stationary phases (CSPs), including those based on saccharides, macrocycles, and porous organic materials [9]. Among these, protein‐based CSPs represent a more specialized category. Human serum albumin (HSA) is a protein‐based CSP that has been successfully employed for both enantiomeric resolution and the evaluation of drug/enantiomer plasma distribution [10, 11]. One of the main advantages of HSA‐based LC is that, unlike many saccharide‐ and macrocycle‐derived CSPs, it is performed in reversed‐phase mode. Other forms are typically operated under normal‐phase conditions and require large volumes of organic solvents, such as *n*‐hexane. The use of HSA results, thus, in a more environmentally sustainable approach due to the use of water‐rich mobile phases.

Chiral recognition by HSA arises from the presence of multiple allosteric binding sites; among these, Sudlow's site I (which binds warfarin) and Sudlow's site II (which binds diazepam) are the most extensively characterized [12]. Notably, HSA has been employed, in combination with other biomimetic stationary phases, to determine the volume of distribution of enantiomers and their corresponding racemates [10].

Conversely, in chiral CE separation, cyclodextrins (CDs) and their derivatives are by far the most used and efficient chiral selectors [13, 14]. In those cases, the methodology is typically capillary electrokinetic chromatography (EKC) or capillary electrochromatography (CEC) (packed or open‐tubular). This study focuses on the chiral separation of azoles, a class of compounds that have garnered substantial attention due to their diverse applications particularly in the pharmaceutical industry [7, 15]. Azoles are a group of five‐membered heterocyclic compounds containing at least one nitrogen atom and are of significant interest due to their extensive use as antifungal agents, among other therapeutic applications [16]. In recent years, triazole fungicides have become an important category of fungicides with wide applicability in agriculture due to their excellent antifungal activity and relatively low environmental persistence. This family of molecules, considered pesticides, was introduced for the treatment and protection of plants, such as cereals, soybeans, fruits, and vegetables, already in the mid‐1970s [17].

The azole family includes imidazoles and triazoles, which have been widely utilized in the treatment of various fungal infections [18]. The unique chemical structure of azoles gives potent antifungal properties for some imidazole derivatives such as ketoconazole (KET), econazole (ECO), and miconazole (MICO) [19], whereas others, like imazalil (IMA) and penconazole (PEN), have fungicidal properties [20]. Many azoles are racemic mixtures due to their chiral structure. Notably, the results of one study indicate that the enantiomers of KET display differentials in their pharmacokinetic profiles in rats [21]. The plasma concentrations of (+)‐KET measured were 2.5 times higher than (−)‐KET, whereas the clearance and volume of distribution of the (−) enantiomer were two‐fold higher than its enantiomer. Further, another study systematically evaluating (+)‐PEN, (−)‐PEN, and (±)‐PEN for liver toxicity in mice showed no adverse effects with the administration of the single (+)‐PEN enantiomer; however, it did show adverse effects on function using (−)‐PEN and (±)‐PEN. Thus, it is essential to treat the enantiomers of chiral drugs as separate medicines requiring full pharmacodynamics and pharmacokinetic profile characterization [22]. By far LC is the most employed methodology for chiral drug analysis, and regarding azoles, several recent papers are available, see, for example, [23, 24, 25] and references therein.

In previous studies, several CDs, including charged and neutral derivatives, have been tested for the chiral separation of antifungal azoles [16, 26, 27, 28, 29, 30]. Recently, Zhou et al. prepared two anionic β‐CD‐based hybrid monolithic phases and applied them to the separation of a set of 27 chiral drugs, including 15 azoles and among them KET, MICO, and IMA [31]. The type of CD‐modified silica monolithic phase had a big impact on the enantioselectivity. Several papers have reported the enantioseparation of KET, MICO, and ECO using 2‐hydroxypropyl‐β‐cyclodextrin (HP‐β‐CD), heptakis(2,3,6‐tri‐*O*‐methyl)‐β‐cyclodextrin (TM‐β‐CD), β‐CD, and sulfobutylether‐β‐CD (SBE‐β‐CD) as chiral selectors [16, 26, 32]. A few papers studied the separation of IMA isomers, using HP‐β‐CD and β‐CD as chiral selectors [33, 34]. Only two studies have been published on the CE separation of PEN. One study reported the chiral separation of PEN using sulfated‐β‐cyclodextrin (S‐β‐CD; degree of substitution 7–11) as the chiral selector [35]. The separation was done at a highly acidic pH value with reversed polarity and with 2% of S‐β‐CD, resulting in the separation of the PEN enantiomers in less than 20 min. Another study used MEKC with sodium dodecyl sulfate (SDS) and 2‐hydroxypropyl‐γ‐CD (HP‐γ‐CD) [36] at highly acidic conditions under reversed polarity for faster (<16 min at 25°C and <13 min at 40°C) enantioseparation of PEN. In that work optimal separations were obtained using a very high HP‐γ‐CD concentration (40 mM), as well as 50 mM SDS and 25 mM phosphate. The current in the capillary was not reported.

With stringent demands placed on the pharmaceutical sector to assess both single stereoisomeric drugs and their racemates, there is a need for more high throughput methodologies to support the advancement of drug development. The present study focuses on the comparison of CDs and HSA as chiral selectors for the separation of a dataset of representative antifungal azoles. Hence, a rapid and efficient EKC–UV/DAD and LC–UV/DAD separation method was developed utilizing low concentrations of chiral selectors. Various CDs were investigated as potential chiral selectors. The findings will illustrate that HSA LC provided limited usefulness in resolving the target azoles, whereas in EKC, HP‐β‐CD was the optimal chiral selector for the enantiomers of ECO, MICO, and IMA. Conversely, the use of TM‐β‐CD was shown to be optimal for the separation of KET, with the aim of achieving the highest resolution within the shortest possible analysis time. Enantioseparation of PEN was for the first time obtained under basic conditions using a dual system chiral selector consisting of a mixture of succinyl‐β‐CD (Succ‐β‐CD) and HP‐β‐CD.

## 2 Materials and Methods

### 2.1 Chemicals and Reagents

Sodium dihydrogen phosphate (NaH_2_PO_4_) was purchased from J.T. Baker (Deventer, Holland). Acetonitrile was obtained from VWR chemicals (Rosny‐sous‐Bois, France). HP‐β‐CD, KET, and MICO were obtained from BLD Pharmatech (Reinbek, Germany), and Succ‐β‐CD (DS = 3.5) was obtained from Cyclolab (Budapest, Hungary). Acetylated‐β‐CD (Ac‐β‐CD, DS = 1) was obtained from American Maize (Stamford, USA). Both 2,6‐di‐*O*‐methyl‐β‐cyclodextrin (DM‐β‐CD) and γ‐CD were purchased from Fluka (Buchs, Switzerland). Orthophosphoric acid, isopropanol (IPA), sodium hydroxide (NaOH) (1 M), and hydrochloric acid (HCl) (1 M) were purchased from Fisher Chemical (Geel, Belgium). ECO was purchased from Thermo Scientific (Loughborough, UK). SDS, methanol (MeOH), sodium tetraborate decahydrate (Borax), sulfated‐β‐CD (S‐β‐CD), TM‐β‐CD, IMA, and PEN were obtained from Sigma Aldrich (St. Louis, USA).

For the LC study, crystalline ammonium acetate (CH_3_COONH_4_), obtained from Alfa Aesar (India) with a purity of ≥97%, was used for buffer preparation. Ammonium hydroxide (NH_4_OH) for pH adjustment was purchased from Merck (Feltham, UK). Standard pH solutions (pH 4.01 and pH 7.00) employed for pH meter calibration were procured from Thermo Scientific (Oxford, UK). (±)‐Warfarin sodium (purity ≥98%) was procured from TRC (Windsor, Connecticut, USA). LC–MS grade acetonitrile and 2‐propanol were purchased from Rathburn Chemicals Ltd. (Walkerburn, Scotland, UK).

### 2.2 Buffer and Sample Preparation

For the CE study sample, stock solutions were made in MeOH at a concentration of 1 mg/mL, except for PEN and IMA, which were prepared at 5 mg/mL. MICO was stored at 4°C, whereas all the other azoles were stored at −20°C to protect against eventual degradation at room temperature. The sample solutions of the analytes were diluted and adjusted in water to the desired concentration in 10% MeOH (v/v). A phosphate running buffer with a concentration of 0.4 M was prepared in water, and the pH was adjusted with orthophosphoric acid to values in the range of 2.7–3.7. Borax buffer was made in water with a concentration of 0.2 M, and HCl (0.1 M) and NaOH (0.1 M) were used to adjust the pH to values between 8.0 and 10.0. All CDs were diluted in water to the final desired concentration. Milli‐Q was generated by a Millipore direct‐Q 3 Milli‐Q water purification system. All solutions were filtered using PTFE 0.45 µm syringe filters before analysis and stored in the fridge or freezer until use. A 7110 InoLab pH meter was used for pH measurement.

For the LC study, the stock azole solutions were prepared to a concentration of 5 mg/mL; the solvent for KET, IMA, and PEN was LC–MS grade methanol, whereas for MICO and ECO it was LC–MS grade ethanol. These solutions were stored at −20°C and used within 3 months. Working solutions were freshly prepared each day by diluting the stock solution to a concentration of 50 µg/mL either with the mobile phase (A) (warfarin, IMA, and PEN) or with LC grade methanol (ECO, MICO, and KET). All samples underwent filtration using 0.45 µm PTFE membrane syringe filters from VWR International (Radnor, USA) before LC analysis. A 20 mM ammonium acetate buffer solution was prepared by dissolving crystalline ammonium acetate (AA) in pure water. Water with a resistivity of 18.2 MΩ/cm was purified in‐house through a Suez water purification system (SUEZ Water Technologies and Solutions). The required pH of the buffer was verified using a Mettler Toledo pH meter (Peterborough, UK) and adjusted with ammonia to either pH 7.00 or pH 5.00. Before each pH adjustment, the pH meter underwent a two‐step calibration, ensuring that the calibration slope remained within a 96% to 104% range. Prior to use, the mobile phase was filtered under vacuum through 0.45 µm nylon membrane filters from Cole‐Parmer. HPLC grade 2‐propanol served as the organic modifier phase without the need for further filtration.

### 2.3 Capillary Electrophoresis

An HPCE G1600AX instrument (Agilent Technologies) equipped with a DAD (*λ* = 190–600 nm) was used. Fused‐silica capillaries from Polymicro Technologies with diameters of 50/360 µm id/od and lengths of 40.0/48.5 cm were used for experiments, unless otherwise specified. Samples were introduced using hydrodynamic pressure with an injection end at the anode at 50 mbar for 5 s. The separation voltage was 20 kV, unless otherwise specified, and the UV detection wavelengths were 200, 210, and 230 nm. The capillary cassette was set at 25°C. To ensure good repeatability, new capillaries were flushed at high pressure (930 mbar) for 15 min with 0.5 M NaOH, for 20 min with Milli‐Q water, and for 10 min with the running buffer. Under acidic conditions, the capillary was flushed (930 mbar) for 2 min with 0.5 M NaOH, followed by a 2 min rinse with 0.1 M HCl, 2 min with Milli‐Q water, and 7 min rinse with the running buffer solution before the first analysis. Between runs, the capillary was rinsed (940 mbar) for 1 min with NaOH (0.5 M), for 1 min with water, and then for 2 min with the running buffer. To enhance both interday and intraday repeatabilities, both electrodes (and capillary ends) were sunk in MeOH solutions for 3 s after each run. For the analysis of PEN under basic conditions, the capillary was rinsed for 5 min with 0.5 M NaOH, followed by a 10 min water rinse at the beginning of the day. Between runs, the capillary was flushed for 2 min with the running buffer.

### 2.4 Liquid Chromatography

HPLC was done on an Agilent 1260 Infinity II high‐performance LC with a 1260 Infinity Diode Array Detector (1290 DAD). The monitored wavelengths were 210, 220, 230, 254, 260, 270, 280, 300, and 310 nm. The analytical column was a Chiralpak HSA 150 mm × 2 mm i.d., 5 µm, and the guard column was Chiralpak 10 mm × 4 mm cartridge, both inserted in a cartridge holder and a column coupler from Daicel Chiral Technologies (Raunheim, Germany). Chromatographic parameters and the sequence analysis were configured using the Microsoft Windows 10 Professional operating PC with Agilent 1290 Infinity 2D‐LC ChemStation software, version 1.4.36. The mobile phases were 20 mM ammonium acetate (A) and 75/25 (v/v) 20 mM ammonium acetate/2‐propanol. The column temperature was set at 30°C, and the injection volume was 5.0 µL. To explore the chiral separation, different LC elution programs were trialed. In Method 1, 100% (A) was applied for 10 min, mobile phase composition was linearly increased to 100% (B) up until 25 min and held for a further 10 min for column decontamination. The mobile phase composition was reverted back to 100% (A) for column re‐equilibration for 14 min. Method 2 was identical except that (B) was held for 30 min rather than 10 min. Method 3 was 100% (A) for 3 min; from 4 to 35 min, the mobile phase composition was set to 100% (B), and then 14 min of column re‐equilibration as before. Method 4 was an isocratic elution program with a mobile phase composition 100% (B), whereas Method 5 was 100% (A) from 0 to 30 min, where the mobile phase composition switched to 100% (B) in 5 min and held for a further 30 min, followed by 14 min of column re‐equilibration at 100% (A). Method 5 was 100% (A) for 30 min, and mobile composition was increased to 100% B in 5 min and held for 30 min, followed by a 20‐min column re‐equilibration at 100% (A). The flow rate was 200 µL/min in all methods. The eluent's pH was either 5.0 or 7.0. Each analyte was analyzed at least in triplicate, and a blank (mobile phase) was injected every six runs to assess any possible carryover effect. All the chromatograms reported are blank subtracted. Resolution was calculated according to the following equation:

(1) $$R_{s}=\frac{1.18\;\left(t_{r2}-t_{r1}\right)}{\left(w_{0.5,1}+w_{0.5,2}\right)}$$

where *t_r_*_1_ and *t_r_*_2_ are the retention times, and *w*_0.5,1,_ and w_0.5,2_ are the widths at half‐height of the first and second chromatographic signals, respectively.

### 2.5 In Silico Calculations

Chemical structures were retrieved from PubChem and imported into MarvinSketch (v22.11, ChemAxon) running on Windows 11. Structures were subjected to 3D cleaning prior to alignment. Three‐dimensional molecular alignments were performed using the built‐in alignment tool with extended atom types, an initial conformation count of two, and accuracy settings set to accurate/normal.

## 3 Results and Discussion

**FIGURE 1:**
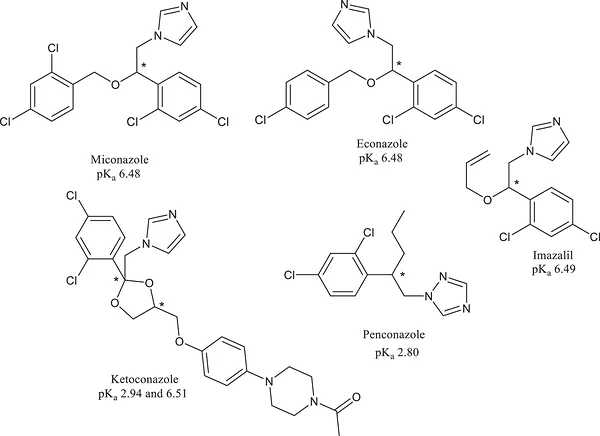
Chemical structures and p*K*_a_ values of the studied azoles. *: Chiral carbons

In this study, HSA and a total of eight CDs were tested as potential chiral selectors using LC and EKC, respectively, for the enantioseparation of five azoles: ECO, MICO, IMA, KET, and PEN. Figure 1 shows the structure of the studied azoles with their respective chiral carbons. This section starts with data on HSA LC–UV/DAD, followed by data on EKC–UV/DAD, including the effects of (a) different types of CDs, (b) CD concentration on the resolution, (c) phosphate concentration in the BGE, (d) pH and voltage on the resolution, (e) dual CD systems for the enantioseparation of PEN, and finally (e) validation.

### 3.1 HSA LC–UV/DAD

The HSA LC column stationary phase is sensitive to denaturing conditions; hence, method development conducted using such columns is rather limited. Specifically, the manufacturer recommends a working pH of between 5 and 7 and a temperature range of 20–30°C for both affinity assessment and chiral separation purposes. The organic modifier ratio, particularly for chiral separation purposes, is restricted to a range of 0%–15% (v/v). The organic modifiers recommended for this column are alcohols rather than nitriles, which are denaturing at comparatively lower concentrations. For this reason, 2‐propanol was selected as an organic modifier as it provides high eluotropic strength and is recommended by the column manufacturer for method scouting.

**FIGURE 2:**
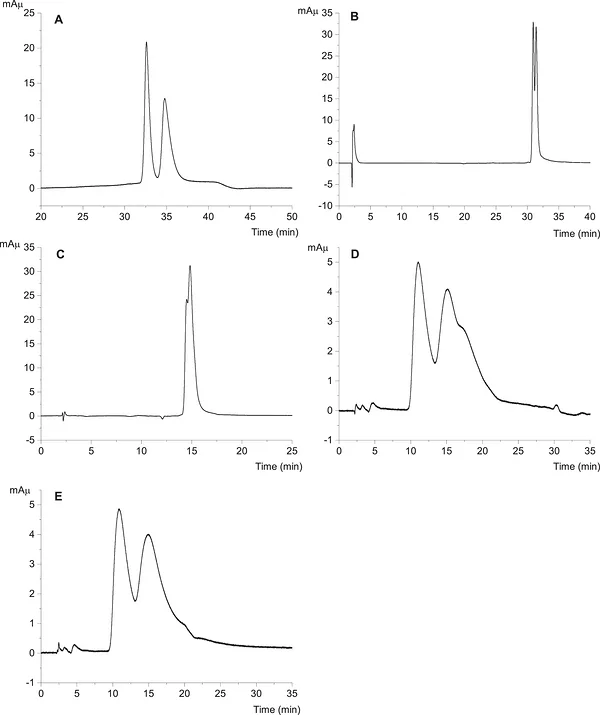
Human serum albumin LC–UV/DAD chromatograms of (±)‐warfarin (A), (±)‐PEN (B and C), and (±)‐IMA (D and E). The mobile phases were 20 mM ammonium acetate (A) and 75/25 (v/v) 20 mM ammonium acetate/2‐propanol (B). The column temperature was 30°C, and the injection volume was 5.0 µL. Chromatograms A, B, and D were obtained using a gradient of 100% A (0–10 min), linearly increased to 100% B (10–25 min), held for 10 min for column washing, followed by 14 min re‐equilibration at 100% A. Chromatogram C used 100% A (0–3 min), 100% B (4–35 min), and 14 min re‐equilibration at 100% A. Chromatogram E used 100% A (0–30 min), a 5 min transition to 100% B, held for 30 min, followed by 14 min re‐equilibration at 100% A (pH 5.0). The eluent pH was 7.0 for A–C and 5.0 for D and E. Detection was at 230 nm for all chromatograms except A (300 nm).

To verify that the enantioselective sites supported on the protein were intact, (±)‐warfarin was selected as a positive control and analyzed before any experimental work was carried out. As could be observed, baseline separation (*R_s_* = 2.11, Figure 2A) was achieved for the enantiomer pair, suggesting that Sudlow's site I on the HSA was intact.

Analysis of the antifungal azole dataset using Method 1 and eluents at pH 7.00 allowed the determination of all the target analytes, excluding MICO and ECO, which are the most lipophilic of the sample set. For this reason, Method 2 was implemented, which allowed the elution of both azoles within 55 min. The logarithms of the chromatographic retention coefficients at both pH values are listed in Table 1. Being these retention indices measured over a linear gradient elution program, their logarithms at pH 7 represent a scale of interaction with the HSA in vivo [37]. Using these methods, PEN was the only azole for which some degree of chiral separation, albeit partial (*R_s_* = 0.66, Figure 2B), occurred. This was to be expected given the HSA has an isoelectric point (pI) of 4.7 [38]. However, both Sudlow sites are rich in cationic residues (arginine, lysine, and histidine), which affords the preferential binding of weak organic acids such as warfarin, salicylates, and many non‐steroidal anti‐inflammatory drugs to the HSA [39]. PEN is the least basic of the azole dataset as it supports a triazole moiety instead of an imidazole ring, which is significantly more alkaline. This feature may have mitigated the electrostatic repulsion between the cationic residues of the Sudlow sites and the studied azoles, which are mostly positively charged at the experimental pH. However, PEN is significantly less ionized at pH 7.00 than the rest of the compounds, given its lower p*K*_a_ value (see Table S1a). Method 3 was applied to reduce the longitudinal diffusion of adjacent peaks by decreasing the isocratic step of both Methods 1 and 2 from 10 to 3 min. Unfortunately, this approach was unsuccessful for this purpose as the only azole for which any degree of chiral separation was observed was PEN, where the two signals were barely distinguishable (*R_s_* = 0.45, Figure 2C), implying that the binding constants of both enantiomers to HSA are of similar values. Both Methods 4 and 5 were unsuccessful; hence, these methods were tested using eluents at pH 5.00 to potentially increase the enantioselectivity of the stationary phase. This is given that most optical resolutions occur in pockets that are very rich in cationic residues where a lower pH will protonate these amino acids at a higher rate. This increased protonation can enhance the cationic nature of the residues present, therefore improving the separation selectivity. Notably, although Sudlow sites I and II are major chiral recognition sites on HSA, enantioseparation can also involve fatty‐acid and secondary binding sites.

**TABLE 1:** Logarithms of chromatographic retention coefficients on the human serum albumin (HSA) phase measured at both pH 5.00 and 7.00 using Method 2.

	log k	
Analyte	pH 7.00	pH 5.00
ECO	1.268	1.133
IMA enantiomer 1	1.108	0.584
IMA enantiomer 2	1.108	0.751
KET	1.102	0.957
MIC	1.331	1.195
PEN enantiomer 1	1.099	1.098
PEN enantiomer 2	1.104	1.133

Abbreviations: ECO, econazole; IMA, imazalil; KET, ketoconazole.

Using eluents at pH 5.00, Method 1 allowed a partial resolution solely for IMA (*R_s_* = 0.72, Figure 2D), whereas by implementing Method 3, both IMA and KET exhibited asymmetric, double‐tailed chromatographic peaks, indicating partial stereoselective interactions with the chiral system (for both *R_s_* < 0.65). However, extensive overlap of the contributing stereoisomeric species was observed, and baseline resolution was not achieved. The implementation of Method 5 also partially resolved the enantiomers of IMA (*R_s_* = 0.79, Figure 2E), whereas double and triple‐tailed peaks were obtained for MICO and ECO, and for KET, respectively, reflecting the co‐elution of stereoisomeric species.

Overall, HSA LC provided limited usefulness in separating the model azole racemic mixtures as only partial resolutions were achieved for IMA and PEN. Although for these racemates, enantiomer identification was allowed, the methods fell short in offering enantiomer quantitation. Interestingly, both of the aforementioned azoles feature only one dichlorobenzene moiety and have similar 3D VdW surface areas (see Figure S1a,b and Table S1a,b) [40]; however, although PEN supports a triazole moiety, IMA features an imidazole ring. The noticeable structural similarity of this azole pair may support the ability of these stereoisomers to access a specific binding site on the protein, potentially underpinning the observed enantioselectivity. The other azoles in the dataset may not access the same site or may do so less efficiently due to their inferior molecular similarity.

### 3.2 EKC–UV/DAD

To circumvent poor enantioselectivity of HSA, we opted to employ CDs as alternative chiral selectors. CDs have been successfully used in GC, SFC, CE (including EKC and CEC), and LC for chiral separation purposes, with the latter two modes being the main separation techniques [41]. However, there are major advantages of the use of CE over LC. CE is more efficient, providing faster analyses and method development, along with simplified sample preparation from biological matrices. This is particularly relevant, for instance, where a chiral separation is applied in a therapeutic drug monitoring setting. Hence, in this work CDs were applied as chiral selectors in EKC mode.

The aim was to achieve fast separations with as low concentration of CDs as possible. CDs have previously been employed as chiral selectors for the enantioseparation of azole compounds, and most CE studies have been conducted under acidic conditions to have the azoles as positively charged compounds [27]. Earlier studies have predominantly focused on a limited array of CDs as chiral recognition agents. Consequently, the objective of the current research was to investigate a wider range of CDs, single or mixed, and to determine their effectiveness in reducing analysis times, as well as CD and buffer concentrations, while maintaining satisfactory resolution. The eight selected CDs included native (β‐CD and γ‐CD), neutral (Ac‐β‐CD, DM‐β‐CD, HP‐β‐CD, and TM‐β‐CD), and charged (S‐β‐CD and Succ‐β‐CD) derivatives.

#### 3.2.1 Effect of Different Types of CDs

A variety of CDs were tested in an initial BGE of 40 mM phosphate (pH 3.2) with 8 mM of the chiral selector. The resolutions of each azole with the respective CDs are listed in Table 2. S‐β‐CD was also investigated, but no peaks were observed after 20 min run for any of the azoles tested (excluded from Table 2). One possible explanation is the strong electrostatic attraction interaction between the negatively charged S‐β‐CD and the positively charged analytes, resulting in very long migration times. Only positive polarity was tested. Among all the CDs tested, neutral HP‐β‐CD was the most effective CD as it had an impact on the separation of four of the five azoles studied. However, the resolution of the KET enantiomers was almost three times higher using neutral TM‐β‐CD, and the migration times were half the ones obtained with HP‐β‐CD. Hence, TM‐β‐CD was selected as the optimal chiral selector for KET, whereas for the three other azoles, HP‐β‐CD was chosen for further experiments. Although a wider range of CDs was tested in this study, the results align with those found in previous studies, where only three different CDs were investigated [32]. As none of the tested CDs resulted in enantiomer separation of PEN, we further investigated the effect of pH (see Section 3.2.4) and the potential benefits of using a dual CD system (see Section 3.2.5) to improve the chiral separation.

**TABLE 2:** Effect of the type of cyclodextrin (CD) on the enantioseparation of the studied azoles.

	β‐CD	HP‐β‐CD	Ac‐β‐CD	γ‐CD	TM‐β‐CD	DM‐β‐CD	Succ‐β‐CD
**ECO**	0.67	0.96	—	0.66	—	—	0.55
**MICO**	—^a^	0.85	—	—	1.19	—	0.93
**IMA**	1.73	2.49	0.96	1.13	1.79	0.76	2.00
**KET**	—	0.60	0.38	—	1.69	0.86	—
**PEN**	—	—	—	—	—	—	—

*Note*: BGE: 40 mM phosphate buffer (pH 3.2) with 8 mM CDs; fused silica capillary: 48.5/40.0 cm total/effective length, 50/360 µm id/od; separation voltage, 20 kV; injection, 50 mbar for 5 s; temperature, 25°C; detection at 200 nm.

Abbreviations: DM‐β‐CD, 2,6‐di‐*O*‐methyl‐β‐cyclodextrin; ECO, econazole; HP‐β‐CD, 2‐hydroxypropyl‐β‐cyclodextrin; IMA, imazalil; KET, ketoconazole; MICO, miconazole; PEN, penconazole; Succ‐β‐CD, succinyl‐β‐CD; TM‐β‐CD, heptakis(2,3,6‐tri‐*O*‐methyl)‐β‐cyclodextrin.

^a^ No separation.

#### 3.2.2 Effect of CD Concentration on the Resolution

**FIGURE 3:**
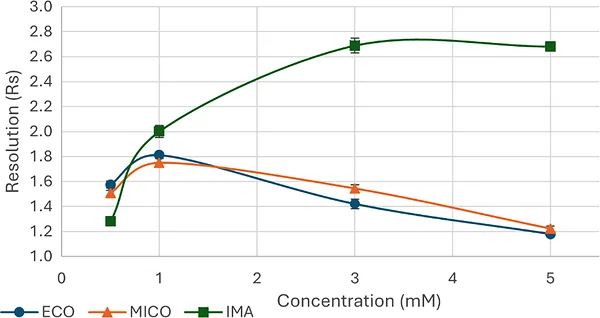
Effect of HP‐β‐CD concentration on ECO, MICO, and IMA enantioseparations. BGE: 40 mM phosphate buffer at pH 3.2 with 0.5–5 mM HP‐β‐CD. Experimental conditions: fused silica capillary: 48.5/40.0 cm total/effective length, 50/360 µm id/od; separation voltage, 20 kV; injection, 50 mbar for 5 s; temperature, 25°C; detection at 200 nm. ECO, econazole; IMA, imazalil; MICO, miconazole.

From the preliminary tests, HP‐β‐CD and TM‐β‐CD showed the biggest impact on the enantiomer separation of the azoles (Table 2). The influence of the concentration of HP‐β‐CD was first tested in the range of 0.5 to 5 mM in 40 mM phosphate at pH 3.2 for ECO, MICO, and IMA (Figures S2–S4, respectively). Figure 3 shows the different effects of HP‐β‐CD on the resolution of the three azoles. For both MICO and ECO, the resolution increased to 1.75 and 1.81, respectively, with an increase in the HP‐β‐CD concentration from 0.5 to 1 mM, but a higher CD concentration led to a significant decrease in the resolution. On the other hand, for IMA, the highest resolution (2.7) was obtained at 3 mM HP‐β‐CD concentration before slowly starting to decrease at higher CD concentrations.

The concentration of TM‐β‐CD on the resolution of the KET enantiomers was tested in the range of 2–10 mM in the same initial buffer as the other azoles (Figure S5). The higher the concentration of the chiral selector, the higher the resolution; however, the longer the migration times. As 8 mM TM‐β‐CD offered a resolution higher than 1.5, with a fast migration time (6 min), it was chosen as the optimal TM‐β‐CD concentration.

#### 3.2.3 Effect of Phosphate Concentration

The effect of increasing the buffer concentration from 20 to 80 mM was studied, with the respective optimal chiral selector concentration for each azole (Table 3). The resolution increased with an increase of phosphate concentration from 1.3 to 2.3, 1.4 to 2.5, 1.9 to 3.2, and 1.3 to 2.5 for ECO, MICO, IMA, and KET, respectively (Figures S6–S9). However, both the current and the migration times increased with higher phosphate concentrations. From 20 to 80 mM of phosphate buffer, the current increased from 16 to 60 µA. To minimize Joule heating, 40 mM of phosphate buffer was chosen for ECO, MICO, and KET and 20 mM for IMA, which were the lowest concentrations at which the resolution was above 1.5 in a short analysis time.

**TABLE 3:** Effect of phosphate buffer concentration on migration times (*n* = 3) and resolution (*R_s_*) values.

	ECO			MICO			IMA			KET		
Conc. (mM)	*t*_1_ ^a^ (min)	*t*_2_ ^b^ (min)	*R_s_*	*t*_1_ (min)	*t*_2_ (min)	*R_s_*	*t*_1_ (min)	*t*_2_ (min)	*R_s_*	*t*_1_ (min)	*t*_2_ (min)	*R_s_*
20	5.20	5.29	1.34	5.11	5.22	1.36	4.96	5.18	1.88	4.92	5.02	1.26
40	6.56	6.72	1.81	6.95	7.14	1.75	6.34	6.68	2.69	5.91	6.05	1.82
60	7.71	7.92	2.11	8.07	8.33	2.23	7.74	8.25	3.11	7.50	7.73	2.56
80	8.03	8.26	2.32	8.55	8.84	2.53	7.46	7.93	3.22	7.78	8.02	2.67

*Note*: BGE: 20–80 mM phosphate buffer (pH 3.2) with 1 mM of HP‐β‐CD for MICO and ECO, 3 mM of HP‐β‐CD for IMA, and 8 mM of TM‐β‐CD for KET; fused silica capillary: 48.5/40.0 cm total/effective length, 50/360 µm id/od; separation voltage, 20 kV; injection, 50 mbar for 5 s; temperature, 25°C; detection at 200 nm.

Abbreviations: ECO, econazole; HP‐β‐CD, 2‐hydroxypropyl‐β‐cyclodextrin; IMA, imazalil; KET, ketoconazole; MICO, miconazole.

^a^ Migration time of peak 1.

^b^ Migration time of peak 2.

#### 3.2.4 Effect of pH and Voltage on the Resolution

The pH affects the charge of the analytes (dissociation/protonation) as well as the magnitude of the EOF and can therefore have a significant effect on the resolution. At acidic pH values (pH <4), all azoles are positively charged. The effect of pH on the resolution was investigated in the range of 2.8–3.7 (Table S2), using phosphate buffer and HP‐β‐CD as the chiral selector. The results show that the more acidic the pH, the longer the migration time, due to a slower EOF, and the higher the resolution of all azoles. As pH 3.2 gave a satisfactory resolution (>1.5) and short migration times for ECO, MICO, and IMA, it was chosen as the optimal pH value.

The pH of the running buffer for KET was also tested (Table S2), and like with the other three azoles, a lower pH resulted in better resolution and longer migration times. As pH 3.2 gave satisfactory resolution and migration times using TM‐β‐CD as a chiral selector, it was selected as the optimal pH of the running buffer.

**FIGURE 4:**
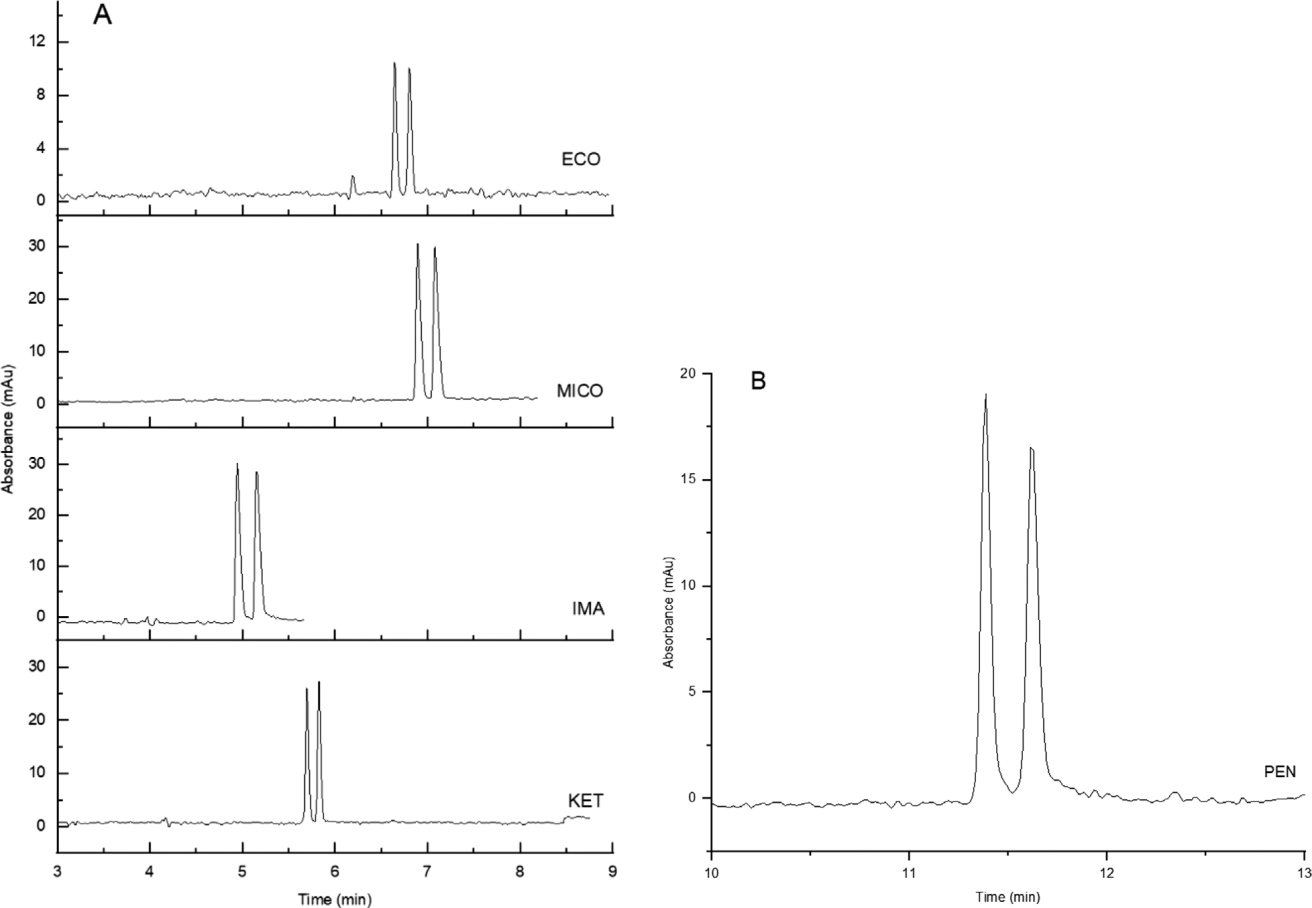
(A) Electropherogram of the enantioseparation of MICO, ECO, IMA, ECO, and KET under optimal conditions. BGE: 40 mM phosphate buffer (pH 3.2) with 1 mM HP‐β‐CD for MICO and ECO and 8 mM TM‐β‐CD for KET; 20 mM phosphate buffer with 3 mM HP‐β‐CD for IMA; fused silica capillary: 48.5/40.0 cm total/effective length, 50/360 µm id/od; separation voltage, 20 kV; injection, 50 mbar for 5 s; temperature, 25°C; detection at 200 nm. (B) Electropherogram of PEN under optimal conditions. BGE: 40 mM borax buffer (pH 9.4) with 2.5 mM HP‐β‐CD and 6 mM Succ‐β‐CD; fused silica capillary: 53.5/45.0 cm total/effective length, 50/360 µm id/od; separation voltage, 12 kV; injection, 50 mbar for 5 s; temperature, 25°C; detection at 200 nm. ECO, econazole; IMA, imazalil; KET, ketoconazole; MICO, miconazole.

The effect of the separation voltage was investigated in the range of 15–25 kV. A decrease in the voltage resulted in increased resolution and longer migration time (see Table S3). A separation voltage of 20 kV resulted in the best balance in terms of short migration time and satisfactory resolution and was employed in all the analyses. Therefore, the optimal BGE conditions were 40 mM phosphate buffer (pH 3.2) containing 1 mM of HP‐β‐CD for ECO and MICO. For IMA, 20 mM of phosphate buffer with 3 mM of HP‐β‐CD offered good resolution. For KET, the optimal BGE was composed of 8 mM of TM‐β‐CD in 40 mM of phosphate buffer (pH 3.2). As shown in Figure 4A, baseline resolution was achieved for all azoles in less than 10 min with a satisfactory resolution (>1.5).

Previously, the separation of ECO, MICO, and KET was studied by Castro‐Puyana et al. [32]. They reported an optimal buffer containing 100 mM phosphate (pH 3.0), with 20 mM of TM‐β‐CD for KET, 2 mM HP‐β‐CD for ECO, and 1 mM HP‐β‐CD for MICO. In their work, the separation voltage was 30 kV and the total capillary length 48.5 cm. The high phosphate buffer concentration (100 mM) in combination with a high electric field strength (619 V/cm) probably resulted in very high current values, and consequently Joule heating. In our work, the concentrations of both the phosphate buffer and the chiral selectors were half or more than half of the ones used in the work by Castro‐Puyana et al. Despite that, in our experiments with a phosphate buffer concentration of 80 mM and an applied voltage of 20 kV, the current was already reaching 60 µA.

#### 3.2.5 Dual CD Systems for the Enantioseparation of PEN

As the preliminary tests showed, none of the CDs demonstrated any chiral separation of the PEN enantiomers under acidic conditions. Therefore, the effect of basic pH values was tested, under which conditions PEN is more likely to be neutral. Dual system CDs containing one anionic and one neutral CD have shown great potential toward the enantioseparation of neutral analytes [42]. In our work, borax buffer was selected over phosphate buffer. With a p*K*_a_ of 9.24, borax provides an effective buffering capacity around 8.0–10.0 [43, 44, 45], whereas that of phosphate is ca. 6–8.5 (p*K*_a2_ 7.21) and ca. 10.5–12 (p*K*_a3_ 12.32). The pH was tested in the range of 8.0–10.0, with the optimal pH determined to be 9.4 (see Table S2). Therefore, under these conditions, borax was a suitable choice for the experiment.

Hence, a dual CD system was tested in an initial buffer of 40 mM of borax (pH 9.4) containing 4 and 10 mM of anionic CD and neutral CD, respectively (see Table 4). First, the negatively charged and neutral CDs were screened to determine the optimal dual system with the highest enantioseparation power. Three different anionic CDs were tested in combination with neutral HP‐β‐CD. The results in Table 4 show that Succ‐β‐CD exhibited higher resolution than Ac‐β‐CD and S‐β‐CD, and therefore, it was tested in combination with five neutral CDs. The dual CD system composed of Succ‐β‐CD and HP‐β‐CD resulted in the highest resolution and showed great potential for the enantioseparation of PEN.

**TABLE 4:** Effect of various cyclodextrins (CDs) in a dual system on the chiral resolution of penconazole (PEN).

Cyclodextrins		*R_s_*
**HP‐β‐CD (a)**	**Succ‐β‐CD**	0.67
	**S‐β‐CD**	0.33
	**Ac‐β‐CD**	—
		
**Succ‐β‐CD (b)**	**HP‐β‐CD**	0.67
	**DM‐β‐CD**	0.33
	**TM‐β‐CD**	0.54
	**γ‐CD**	—
	**β‐CD**	0.53

*Note*: BGE: 40 mM borax buffer at pH 9.4 with (a) 10 mM HP‐β‐CD and 4 mM anionic CD, (b) 4 mM Succ‐β‐CD and 10 mM neutral CD; fused silica capillary: 48.5/40.0 cm total/effective length, 50/360 µm id/od; separation voltage, 20 kV; injection, 50 mbar for 5 s; temperature, 25°C; detection at 200 nm.

Abbreviations: HP‐β‐CD, 2‐hydroxypropyl‐β‐cyclodextrin; Succ‐β‐CD, succinyl‐β‐CD; TM‐β‐CD, heptakis(2,3,6‐tri‐*O*‐methyl)‐β‐cyclodextrin; DM‐β‐CD, 2,6‐di‐*O*‐methyl‐β‐cyclodextrin.

The effect of borax concentration was first investigated in an initial buffer (pH 9.4) containing 4 mM of Succ‐β‐CD and 5 mM of HP‐β‐CD. Increasing the borax concentration from 20 to 80 mM had a minimal impact on the resolution, ranging from 0.80 to 0.90, with the highest resolution and longest migration time observed with 80 mM of borax (Figure S10). However, this concentration led to a high current (>100 µA). Given that the resolution difference between 40 and 80 mM was minimal (0.87–0.90), 40 mM was chosen as the optimal concentration to minimize Joule heating and ensure faster migration times. The concentrations of both chiral selectors were examined using the selected borax concentration of 40 mM (pH 9.4). By keeping one of the chiral selector concentrations constant while changing the other, we were able to determine the optimal concentration of both CDs. Variations in the Succ‐β‐CD concentration had a greater impact on the resolution than HP‐β‐CD. Reducing the neutral CD concentration to 2.5 mM led to an increase in the resolution, whereas increasing the Succ‐β‐CD concentration up to 6 mM resulted in an increase in the resolution, after which it gradually decreased (Table S4).

**FIGURE 5:**
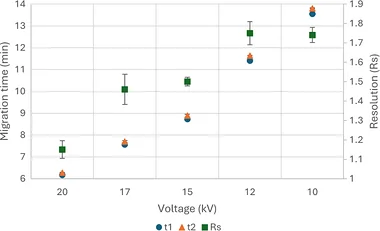
Effect of separation voltage on resolution and migration times of PEN enantiomers. BGE: 40 mM borax buffer (pH 9.4) with 2.5 mM HP‐β‐CD and 6 mM Succ‐β‐CD; fused silica capillary: 53.5/45.0 cm total/effective length, 50/360 µm id/od; separation voltage, 10–20 kV; injection, 50 mbar for 5 s; temperature, 25°C; detection at 200 nm.

By optimizing the buffer concentration, the highest resolution obtained for the PEN enantiomers was 0.94, which is still considered a low resolution. Other parameters such as the addition of a surfactant (SDS) and organic solvents (MeOH, ACN, IPA) to the BGE were tested (data not shown); however, they did not affect the resolution but, on the contrary, decreased it. Therefore, a longer capillary was tested. The longer the capillary, the longer the migration times, and typically, the better the resolution. By increasing the effective capillary length from 40 to 45 cm, the resolution increased from 0.9 to 1.2. As the resolution was still too low, the separation voltage was investigated in the range of 20–10 kV. The results in Figure 5 show that the resolution increased with a decrease in the separation voltage. However, as the resolution remained unchanged between 10 and 12 kV, but the lower voltage led to longer migration times, 12 kV was chosen as the optimal separation voltage, ensuring a resolution greater than 1.7 while keeping the analysis time under 12 min. Furthermore, by increasing the capillary length and decreasing the voltage, the current decreased from 47 to 23 µA, minimizing Joule heating. Figure 4B shows the electropherogram of the enantiomeric separation of PEN under optimal BGE conditions.

#### 3.2.6 Validation of the EKC Method

The EKC method was validated with respect to linearity, LOD, LOQ, repeatability, and precision. The linearity of the method was studied in the respective range of each compound (Table S5). Triplicate injections at six concentrations were performed. Linear regression equations were obtained by plotting the peak area versus the concentration of each azole. The analysis resulted in high determination coefficients (*R* ^2^ > 0.963) for all azoles. The LOD and LOQ were determined at a signal‐to‐noise ratio of 3:1 and 10:1, respectively (Table S5). The LODs were in the range of 6–9 µg/mL and the LOQs in the range of 13–20 µg/mL. The lowest LOD values were seen for IMA and PEN, whereas the lowest LOQ value was seen for PEN. Previous studies on CD‐EKC have reported LOD values in the range of 0.7–3.6 µg/mL for ECO [46, 47], 0.1–0.26 µg/mL for IMA [33, 48], 0.075–0.25 µg/mL for KET [26, 49], and 0.075–20 µg/mL for MICO [26, 45]. The only previously reported LOD value for PEN is by CD‐MECK, giving a value of 4.1 µg/mL [36]. LOQ values have typically not been reported; however, they have been determined in a few studies, with values of 0.25 µg/mL for KET and MICO [26], and in the range of 2.2–12.1 µg/mL for ECO [46, 47]. However, to note here is that in the current study, we did not further optimize the methodologies for reaching even lower LODs or LOQs, for example, by online stacking methodologies [50, 51, 52, 53], but instead we wanted to report the values that we got using our methods, which were optimized by means of the separation resolution.

The intraday and interday repeatabilities, expressed in terms of RSD values, were evaluated to investigate the precision of the method (Table S6). The intraday precision was obtained by performing six consecutive injections of each azole under their respective optimal BGE conditions within a single day. The intraday repeatability (*n *= 6) RSD values ranged from 0.4% to 1.1% for the migration times, from 1.7% to 10.6% for the peak areas, and from 1.8% to 10.9% for the migration time‐corrected areas, indicating satisfactory precision. Interday repeatability was tested for some of the compounds by performing six injections on two consecutive days. The interday repeatability (*n* = 12) RSD values ranged from 1.6% to 1.9% for the migration times, from 3.9% to 20.5% for the peak areas, and from 3.0% to 22.0% for the migration time‐corrected areas, with ECO showing the highest RSD values.

## 4 Concluding Remarks

In this work, methods employing LC‐UV/DAD based on HSA and CD‐EKC–UV/DAD based on CDs were developed for the enantiomeric separation of five selected pharmaceutical and agrochemical azole derivatives. While useful to assess HSA binding affinity of antifungal azoles, HSA LC allowed for the partial resolution of only two out of five racemates, namely, for IMA and PEN. These azoles feature a high degree of similarity (similar surface area and volumes), which may indicate the preferential access of these stereoisomers to the same enantioselective pocket on HSA. Consequently, efforts were directed toward CE using CDs as chiral selectors.

In the first part of the EKC study, two different CDs were used, with TM‐β‐CD identified as the most suitable chiral selector for KET, whereas HP‐β‐CD was selected for ECO, MICO, and IMA. Under acidic conditions (pH of 3.2), a high resolution (*R_s_ *> 1.8) was obtained with an analysis time of less than 8 min for all four azoles. The optimal CD concentrations were in the range of 1–8 mM. In the second part of the study, the chiral separation of PEN enantiomers was achieved by introducing a dual CD system. Under basic conditions (pH 9.4), PEN enantiomers were successfully separated with 40 mM borax buffer containing low concentrations of the chiral selectors; 2.5 mM of HP‐β‐CD and 6 mM of Succ‐β‐CD. By combining an anionic CD (Succ‐β‐CD) with a neutral CD (HP‐β‐CD), high enantioresolution (>1.7) was achieved in a short analysis time (11 min), demonstrating the effectiveness of a dual CD system over a single CD system for PEN. The LODs and LOQs for the compounds studied were in the range of 6–9 and 13–20 µg/mL, respectively, with the lowest values seen for PEN. The EKC separations using the optimal BGEs were fast and repeatable, and the developed methods were cost‐effective as only low amounts of CDs were required.

As demonstrated in this work, HSA‐LC showed limited selectivity for the investigated azoles, whereas CD‐based EKC provided more effective enantioseparations under the studied conditions. The future perspectives of the developed methodologies include adopting green chemistry approaches by further reducing the amounts of CDs, optimizing buffer systems to speed up analyses, and minimizing waste. The implementation of such novel methodologies aims to enhance environmental sustainability within the pharmaceutical sector to align with green chemistry principles, while maintaining the ability to create well‐resolved safety profiles of racemates in drug development and manufacturing for quality control and regulatory compliance purposes.

## Author Contributions

**Sarah Moreau**: investigation, visualization, writing – original draft, writing – review and editing. **Amy Dillon**: writing – original draft, writing – review and editing. **Giacomo Russo**: investigation, writing – original draft, writing – review and editing. **Susanne K. Wiedmer**: conceptualization, funding acquisition, project administration, supervision, writing – original draft, writing – review and editing.

## Funding

Financial support from the Finnish Society of Sciences and Letters under grant number 4707794 (SKW), from the Swedish Cultural Foundation under grant number 4707795 (SKW), and from the Waldemar von Frenckell's foundation under grant number 4710098 (SKW) are acknowledged.

## Conflicts of Interest

The authors declare no conflicts of interest.

## Supporting information



Additional material (Figures S1‐S10 and Tables S1‐S6) can be found online in the Supporting Information section.**Supporting File**: elps70124‐sup‐0001‐SuppMat.docx.

## Data Availability

The data that support the findings of this study are available from the corresponding author upon reasonable request.
