# Supplementary material for: Protein‐Based High‐Performance Liquid Chromatography and Cyclodextrin‐Capillary Electrokinetic Chromatography for the Chiral Separation of Azoles
Source: Electrophoresis. 2026 Jun 23;47(9):813–25. doi: 10.1002/elps.70124 (PMC13572895; doi:10.1002/elps.70124)
Supplement: Supplementary file 1 [file ELPS-47--s001.docx]

**Supporting material**

**Protein-based high-performance liquid chromatography and cyclodextrin-capillary electrokinetic chromatography for the chiral separation of azoles**

Sarah Moreau^1^, Amy Dillon^2^, Giacomo Russo^2^, Susanne K. Wiedmer^1*^

^1^ Department of Chemistry, A.I. Virtasen aukio 1, P. O. Box 55, 00014 University of Helsinki, Finland

^2^ Centre for Biomedicine and Global Health, School of Applied Sciences, Sighthill Campus, Edinburgh Napier University, 9 Sighthill Ct, EH11 4BN Edinburgh, United Kingdom

**Figure captions**

**Figure S1a.** Three-dimensional conformer overlaps for the azole pairs PEN-IMA, ECO-MICO, ECO-KET, ECO-PEN, and MICO-IMA. Structural alignment was performed using the 3D Alignment plugin implemented in Marvin Sketch (ChemAxon), highlighting the spatial correspondence and conformational similarity between each azole pair.

**Figure S1b.** Three-dimensional conformer overlaps for the azole pairs MICO-KET, MICO-PEN, IMA-KET, KET-PEN, and ECO-IMA. Structural alignment was performed using the 3D Alignment plugin implemented in Marvin Sketch (ChemAxon), highlighting the spatial correspondence and conformational similarity between each azole pair.

**Figure S2.** Electropherograms of the effect of HP-β-CD concentration on ECO enantioseparation. BGE: 40 mM phosphate buffer (pH 3.2) with 0.5-5 mM HP-β-CD; fused silica capillary: 48.5/40.0 cm total/effective length, 50/360 μm id/od; separation voltage, 20 kV; injection, 50 mbar for 5 s; temperature 25 °C; detection at 200 nm.

**Figure S3**. Electropherograms of the effect of HP-β-CD concentration on MICO enantioseparation. BGE: 40 mM phosphate buffer (pH 3.2) with 0.5-5 mM HP-β-CD; fused silica capillary: 48.5/40.0 cm total/effective length, 50/360 μm id/od; separation voltage, 20 kV; injection, 50 mbar for 5 s; temperature 25 °C; detection at 200 nm.

**Figure S4.** Electropherograms of the effect of HP-β-CD concentration on IMA enantioseparation. BGE: 40 mM phosphate buffer (pH 3.2) with 0.5-5 mM HP-β-CD; fused silica capillary: 48.5/40.0 cm total/effective length, 50/360 μm id/od.; separation voltage, 20 kV; injection, 50 mbar for 5 s; temperature 25 °C; detection at 200 nm.

**Figure S5.** Electropherograms of the effect of TM-β-CD concentration on KET enantioseparation. BGE: 40 mM phosphate buffer (pH 3.2) with 2-10 mM TM-β-CD; fused silica capillary: 48.5/40.0 cm total/effective length, 50/360 μm id/od; separation voltage, 20 kV; injection, 50 mbar for 5 s; temperature 25 °C; detection at 200 nm.

**Figure S6.** Electropherograms of ECO enantiomer resolution at different phosphate buffer concentrations. BGE: 20-80 mM phosphate buffer (pH 3.2) with 1 mM HP-β-CD; fused silica capillary: 48.5/40.0 cm total/effective length, 50/360 μm id/od; separation voltage, 20 kV; injection, 50 mbar for 5 s; temperature 25 °C; detection at 200 nm.

**Figure S7.** Electropherograms of MICO enantiomer resolution at different phosphate buffer concentrations. BGE: 20-80 mM phosphate buffer (pH 3.2) with 1 mM HP-β-CD; fused silica capillary: 48.5/40.0 cm total/effective length, 50/360 μm id/od; separation voltage, 20 kV; injection, 50 mbar for 5 s; temperature 25 °C; detection at 200 nm.

**Figure S8.** Electropherograms of IMA enantiomer resolution at different phosphate buffer concentrations. BGE: 20-80 mM phosphate buffer (pH 3.2) with 3 mM HP-β-CD; fused silica capillary: 48.5/40.0 cm total/effective length, 50/360 μm id/od; separation voltage, 20 kV; injection, 50 mbar for 5 s; temperature 25 °C; detection at 200 nm.

**Figure S9.** Electropherograms of KET enantiomer resolution at different phosphate buffer concentrations. BGE: 20-80 mM phosphate buffer (pH 3.2) with 8 mM TM-β-CD; fused silica capillary: 48.5/40.0 cm total/effective length, 50/360 μm id/od; separation voltage, 20 kV; injection, 50 mbar for 5 s; temperature 25 °C; detection at 200 nm.

**Figure S10.** Electropherograms of the enantioseparation of PEN with an increase in borax concentration; 20 to 80 mM. BGE: 20-80 mM borax buffer (pH 9.4) with 2.5 mM HP-β-CD and 6 mM Succ-β-CD; fused silica capillary: 48.5/40.0 cm total/effective length, 50/360 μm id/od; separation voltage, 12 kV; injection, 50 mbar for 5 s; temperature 25 °C; detection at 200 nm.

**Table captions**

**Table S1a**. Molecular masses, log P^N^, log D^7.4^, pK_a_ and Van der Waals surface areas and volumes of the azoles considered in this study. The calculations were based on MarvinSketch 22.11 (ChemAxon).

**Table S1b**. 3D similarity coefficients using according to the 3D alignment plug in using two accuracy options (normal and accurate). The calculations were based on MarvinSketch 22.11 (ChemAxon).

**Table S2.** Effect of pH on the resolution and migration times (n=3) of the studied azoles under optimal conditions. t_1_ and t_2_ stand for the enantiomers. BGE: 40 mM phosphate buffer with 1 mM HP-β-CD for MICO and ECO, 20 mM phosphate buffer with 3 mM HP-β-CD for IMA, 60 mM phosphate buffer with 18 mM HP-β-CD for KET, 40 mM borax buffer with 2.5 mM HP-β-CD and 6 mM Succ-β-CD for PEN; fused silica capillary: 48.5/40.0 cm total/effective length, 50/360 μm id/od; separation voltage, 20 kV; injection, 50 mbar for 5 s; temperature 25 °C; detection at 200 nm.

**Table S3.** Effect of separation voltage on resolution and migration times (n=3) of the studied azoles under optimal conditions. BGE: 40 mM phosphate buffer with 1 mM HP-β-CD for MICO and ECO, 20 mM phosphate buffer with 3 mM of HP-β-CD for IMA; fused silica capillary: 48.5/40.0 cm total/effective length, 50/360 μm id/od; separation voltage, 15-25 kV; injection, 50 mbar for 5 s; temperature 25 °C; detection at 200 nm.

**Table S4.** Effect of Succ-β-CD and HP-β-CD on the resolution (n=3) of PEN enantiomers. BGE: 40 mM borax buffer (pH 9.4) with 2.5-10 mM HP-β-CD and 4.0-10.0 mM Succ-β-CD; fused silica capillary: 48.5/40.0 cm total/effective length, 50/360 μm id/od; separation voltage, 20 kV; injection, 50 mbar for 5 s; temperature 25 °C; detection at 200 nm.

**Table S5.** Validation data of developed method for the enantioseparation of azoles. BGE: 40 mM phosphate buffer (pH 3.2) with 1 mM HP-β-CD for MICO and ECO and 8 mM TM-β-CD for KET, 20 mM phosphate buffer with 3 mM HP-β-CD for IMA; Borax buffer at pH 9.4, 2.5 mM HP-β-CD and 6 mM Succ-β-CD for PEN; fused silica capillary: 48.5/40.0 cm total/effective length (53.5/50.0 cm for PEN), 50/360 μm id/od; separation voltage, 20 kV (12 kV for PEN); injection, 50 mbar for 5 s; temperature 25 °C; detection at 200 nm.

**Table S6.** Repeatability (intraday and interday) of the enantiomeric separation of the azoles. The enantiomers are marked as t_1_ and t_2_. BGE: 40 mM phosphate buffer (pH 3.2), 1 mM HP-β-CD for MICO and ECO, 8 mM TM-β-CD for KET, and 20 mM phosphate buffer with 3 mM HP-β-CD for IMA. Borax buffer at pH 9.4 with 2.5 mM HP-β-CD and 6 mM Succ-β-CD for PEN; fused silica capillary: 48.5/40.0 cm total/effective length (53.5/50.0 cm for PEN), 50/360 μm id/od; separation voltage, 20 kV (12 kV for PEN); injection, 50 mbar for 5 s; temperature 25 °C; detection at 200 nm.

**Figure S1a.** Three-dimensional conformer overlaps for the azole pairs PEN-IMA, ECO-MICO, ECO-KET, ECO-PEN, and MICO-IMA. Structural alignment was performed using the 3D Alignment plugin implemented in Marvin Sketch (ChemAxon), highlighting the spatial correspondence and conformational similarity between each azole pair.

**Figure S1b.** Three-dimensional conformer overlaps for the azole pairs MICO-KET, MICO-PEN, IMA-KET, KET-PEN, and ECO-IMA. Structural alignment was performed using the 3D Alignment plugin implemented in Marvin Sketch (ChemAxon), highlighting the spatial correspondence and conformational similarity between each azole pair.

**Figure S2.** Electropherograms of the effect of HP-β-CD concentration on ECO enantioseparation. BGE: 40 mM phosphate buffer (pH 3.2) with 0.5-5 mM HP-β-CD; fused silica capillary: 48.5/40.0 cm total/effective length, 50/360 μm id/od; separation voltage, 20 kV; injection, 50 mbar for 5 s; temperature 25 °C; detection at 200 nm.

**Figure S3.** Electropherograms of the effect of HP-β-CD concentration on MICO enantioseparation. BGE: 40 mM phosphate buffer (pH 3.2) with 0.5-5 mM HP-β-CD; fused silica capillary: 48.5/40.0 cm total/effective length, 50/360 μm id/od; separation voltage, 20 kV; injection, 50 mbar for 5 s; temperature 25 °C; detection at 200 nm.

**Figure S4.** Electropherograms of the effect of HP-β-CD concentration on IMA enantioseparation. BGE: 40 mM phosphate buffer (pH 3.2) with 0.5-5 mM HP-β-CD; fused silica capillary: 48.5/40.0 cm total/effective length, 50/360 μm id/od.; separation voltage, 20 kV; injection, 50 mbar for 5 s; temperature 25 °C; detection at 200 nm.

**Figure S5.** Electropherograms of the effect of TM-β-CD concentration on KET enantioseparation. BGE: 40 mM phosphate buffer (pH 3.2) with 2-10 mM TM-β-CD; fused silica capillary: 48.5/40.0 cm total/effective length, 50/360 μm id/od; separation voltage, 20 kV; injection, 50 mbar for 5 s; temperature 25 °C; detection at 200 nm.

**Figure S6.** Electropherograms of ECO enantiomer resolution at different phosphate buffer concentrations. BGE: 20-80 mM phosphate buffer (pH 3.2) with 1 mM HP-β-CD; fused silica capillary: 48.5/40.0 cm total/effective length, 50/360 μm id/od; separation voltage, 20 kV; injection, 50 mbar for 5 s; temperature 25 °C; detection at 200 nm.

**Figure S7.** Electropherograms of MICO enantiomer resolution at different phosphate buffer concentrations. BGE: 20-80 mM phosphate buffer (pH 3.2) with 1 mM HP-β-CD; fused silica capillary: 48.5/40.0 cm total/effective length, 50/360 μm id/od; separation voltage, 20 kV; injection, 50 mbar for 5 s; temperature 25 °C; detection at 200 nm.

**Figure S8.** Electropherograms of IMA enantiomer resolution at different phosphate buffer concentrations. BGE: 20-80 mM phosphate buffer (pH 3.2) with 3 mM HP-β-CD; fused silica capillary: 48.5/40.0 cm total/effective length, 50/360 μm id/od; separation voltage, 20 kV; injection, 50 mbar for 5 s; temperature 25 °C; detection at 200 nm.

**Figure S9.** Electropherograms of KET enantiomer resolution at different phosphate buffer concentrations. BGE: 20-80 mM phosphate buffer (pH 3.2) with 8 mM TM-β-CD; fused silica capillary: 48.5/40.0 cm total/effective length, 50/360 μm id/od; separation voltage, 20 kV; injection, 50 mbar for 5 s; temperature 25 °C; detection at 200 nm.

**Figure S10.** Electropherograms of the enantioseparation of PEN with an increase in borax concentration; 20 to 80 mM. BGE: 20-80 mM borax buffer (pH 9.4) with 2.5 mM HP-β-CD and 6 mM Succ-β-CD; fused silica capillary: 48.5/40.0 cm total/effective length, 50/360 μm id/od; separation voltage, 12 kV; injection, 50 mbar for 5 s; temperature 25 °C; detection at 200 nm.

**Table S1a**. Molecular masses, log P^N^, log D^7.4^, pK_a_ and Van der Waals surface areas and volumes of the azoles considered in this study. The calculations were based on MarvinSketch 22.11 (ChemAxon).

|  | M.W. (g mol^-1^) | log P^N^ | log D^7.4^ | pK_a_ | VdW SA (Å^2^) | VdW Vol (Å^3^) |
| --- | --- | --- | --- | --- | --- | --- |
| KET | 531.43 | 4.28 | 4.16 | 6.42 | 725.20 | 450.46 |
| MICO | 416.12 | 5.53 | 5.40 | 6.48 | 490.38 | 319.51 |
| ECO | 381.68 | 5.01 | 4.88 | 6.48 | 474.89 | 305.52 |
| IMA | 297.18 | 3.46 | 3.32 | 6.48 | 382.94 | 247.36 |
| PEN | 284.18 | 3.77 | 3.77 | 2.06 | 389.50 | 241.34 |

**Table S1b**. 3D similarity coefficients using according to the 3D alignment plug in using two accuracy options (normal and accurate). The calculations were based on MarvinSketch 22.11 (ChemAxon).

|  | 3D similarity (normal) | 3D similarity (accurate) |
| --- | --- | --- |
| PEN-IMA | 0.780 | 0.695 |
| ECO-MICO | 0.973 | 0.918 |
| ECO-KET | 0.715 | 0.599 |
| ECO-PEN | 0.587 | 0.569 |
| MICO-IMA | 0.606 | 0.470 |
| MICO-KET | 0.701 | 0.565 |
| MICO-PEN | 0.572 | 0.537 |
| IMA-KET | 0.479 | 0.456 |
| KET-PEN | 0.500 | 0.476 |
| ECO-IMA | 0.623 | 0.498 |

**Table S2.** Effect of pH on the resolution and migration times (n=3) of the studied azoles under optimal conditions. t_1_ and t_2_ stand for the enantiomers. BGE: 40 mM phosphate buffer with 1 mM HP-β-CD for MICO and ECO, 20 mM phosphate buffer with 3 mM HP-β-CD for IMA, 60 mM phosphate buffer with 18 mM HP-β-CD for KET, 40 mM borax buffer with 2.5 mM HP-β-CD and 6 mM Succ-β-CD for PEN; fused silica capillary: 48.5/40.0 cm total/effective length, 50/360 μm id/od; separation voltage, 20 kV; injection, 50 mbar for 5 s; temperature 25 °C; detection at 200 nm.

| **pH** | **2.8** | | | **3.0** | | | **3.2** | | | **3.5** | | | **3.7** | | |
| --- | --- | --- | --- | --- | --- | --- | --- | --- | --- | --- | --- | --- | --- | --- | --- |
|  | **t_1_**  **(min)** | **t_2_**  **(min)** | **R_s_** | **t_1_**  **(min)** | **t_2_**  **(min)** | **R_s_** | **t_1_**  **(min)** | **t_2_**  **(min)** | **R_s_** | **t_1_**  **(min)** | **t_2_**  **(min)** | **R_s_** | **t_1_**  **(min)** | **t_2_**  **(min)** | **R_s_** |
| **ECO** | 7.54 | 7.75 | 2.17 | 6.89 | 7.06 | 1.86 | 6.56 | 6.72 | 1.81 | 5.02 | 5.11 | 1.33 | 4.30 | 4.37 | 1.06 |
| **MICO** | 7.93 | 8.18 | 2.40 | 7.21 | 7.42 | 2.10 | 6.95 | 7.14 | 1.80 | 5.22 | 5.33 | 1.47 | 4.07 | 4.14 | 0.90 |
| **IMA** | 6.05 | 6.35 | 2.60 | 5.41 | 5.66 | 2.30 | 4.96 | 5.18 | 1.88 | 3.79 | 3.91 | 1.50 | 3.18 | 3.26 | 1.21 |
| **KET** | 12.21 | 12.41 | 1.50 | 12.07 | 12.26 | 1.41 | 11.62 | 11.77 | 1.08 | 10.16 | 10.25 | 0.85 | 7.86 | -^†^ | - |
|  |  |  |  |  |  |  |  |  |  |  |  |  |  |  |  |
| **pH** | **8.0** | | | **8.6** | | | **9.0** | | | **9.4** | | | **10.0** | | |
|  | **t_1_**  **(min)** | **t_2_**  **(min)** | **R_s_** | **t_1_**  **(min)** | **t_2_**  **(min)** | **R_s_** | **t_1_**  **(min)** | **t_2_**  **(min)** | **R_s_** | **t_1_**  **(min)** | **t_2_**  **(min)** | **R_s_** | **t_1_**  **(min)** | **t_2_**  **(min)** | **R_s_** |
| **PEN** | 5.19 | 5.36 | 0.74 | 5.21 | 5.29 | 0.72 | 5.18 | 5.26 | 0.87 | 4.99 | 5.06 | 0.94 | 5.77 | 5.86 | 0.87 |

^†^) no separation

**Table S3.** Effect of separation voltage on resolution and migration times (n=3) of the studied azoles under optimal conditions. BGE: 40 mM phosphate buffer with 1 mM HP-β-CD for MICO and ECO, 20 mM phosphate buffer with 3 mM of HP-β-CD for IMA; fused silica capillary: 48.5/40.0 cm total/effective length, 50/360 μm id/od; separation voltage, 15-25 kV; injection, 50 mbar for 5 s; temperature 25 °C; detection at 200 nm.

| **Voltage (kV)** | **15** | | | **20** | | | **25** | | |
| --- | --- | --- | --- | --- | --- | --- | --- | --- | --- |
|  | **t_1_**  **(min)** | **t_2_**  **(min)** | **R_s_** | **t_1_**  **(min)** | **t_2_**  **(min)** | **R_s_** | **t_1_**  **(min)** | **t_2_**  **(min)** | **R_s_** |
| **ECO** | 8.42 | 8.61 | 1.94 | 6.56 | 6.72 | 1.81 | 5.50 | 5.63 | 1.65 |
| **MICO** | 8.42 | 8.64 | 1.93 | 6.95 | 7.14 | 1.75 | 4.46 | 4.56 | 1.47 |
| **IMA** | 5.85 | 6.06 | 1.94 | 4.96 | 5.18 | 1.89 | 3.16 | 3.27 | 1.39 |
| **KET** | N/A^†^ | N/A | N/A | N/A | N/A | N/A | N/A | N/A | N/A |

^†^) data not available

**Table S4.** Effect of Succ-β-CD and HP-β-CD on the resolution (n=3) of PEN enantiomers. BGE: 40 mM borax buffer (pH 9.4) with 2.5-10 mM HP-β-CD and 4.0-10.0 mM Succ-β-CD; fused silica capillary: 48.5/40.0 cm total/effective length, 50/360 μm id/od; separation voltage, 20 kV; injection, 50 mbar for 5 s; temperature 25 °C; detection at 200 nm.

| **Conc. (mM)** | | **Resolution** | **Conc. (mM)** | | **Resolution** |
| --- | --- | --- | --- | --- | --- |
| **HP-β-CD** | **Succ-β-CD** | **R_s_** | **HP-β-CD** | **Succ-β-CD** | **R_s_** |
| 10.0 | 4.0 | 0.70 | 2.5 | 4.0 | 0.88 |
| 5.0 | 4.0 | 0.87 | 2.5 | 6.0 | 0.94 |
| 2.5 | 4.0 | 0.88 | 2.5 | 8.0 | 0.90 |
| 0 | 4.0 | 0.15 | 2.5 | 10.0 | 0.78 |

**Table S5.** Validation data of developed method for the enantioseparation of azoles. BGE: 40 mM phosphate buffer (pH 3.2) with 1 mM HP-β-CD for MICO and ECO and 8 mM TM-β-CD for KET, 20 mM phosphate buffer with 3 mM HP-β-CD for IMA; Borax buffer at pH 9.4, 2.5 mM HP-β-CD and 6 mM Succ-β-CD for PEN; fused silica capillary: 48.5/40.0 cm total/effective length (53.5/50.0 cm for PEN), 50/360 μm id/od; separation voltage, 20 kV (12 kV for PEN); injection, 50 mbar for 5 s; temperature 25 °C; detection at 200 nm.

| **Compounds** | **ECO** | **MICO** | **IMA** | **KET** | **PEN** |
| --- | --- | --- | --- | --- | --- |
| **Calibration range (μg/mL)** | 30-80 | 30-90 | 90-40 | 20-60 | 90-40 |
| **Regression equation** | y=0.874x-15.586 | y=1.363x+10.705 | y=1.041x+2.894 | y=1.210x+6.213 | y=1.647x+3.768 |
| **Correlation coefficient** | 0.972 | 0.996 | 0.967 | 0.963 | 0.998 |
| **LOD (μg/mL)** | 9 | 8 | 6 | 9 | 6 |
| **LOQ (μg/mL)** | 20 | 20 | 14 | 20 | 13 |

**Table S6.** Repeatability (intraday and interday) of the enantiomeric separation of the azoles. The enantiomers are marked as t_1_ and t_2_. BGE: 40 mM phosphate buffer (pH 3.2), 1 mM HP-β-CD for MICO and ECO, 8 mM TM-β-CD for KET, and 20 mM phosphate buffer with 3 mM HP-β-CD for IMA. Borax buffer at pH 9.4 with 2.5 mM HP-β-CD and 6 mM Succ-β-CD for PEN; fused silica capillary: 48.5/40.0 cm total/effective length (53.5/50.0 cm for PEN), 50/360 μm id/od; separation voltage, 20 kV (12 kV for PEN); injection, 50 mbar for 5 s; temperature 25 °C; detection at 200 nm.

| %RSD | **ECO** | **MICO** | **IMA** | **KET** | **PEN** |
| --- | --- | --- | --- | --- | --- |
| **Repeatability (intraday, n=6)** |  |  |  |  |  |
| t1 (day 1) | 0.7 | 0.9 | 1.1 | 0.5 | 0.1 |
| t2 (day 1) | 0.7 | 0.9 | 1.2 | 0.5 | 0.1 |
| t1 (day 2) | 1.4 | 1.1 | 1.6 | N/A^†^ | N/A |
| t2 (day 2) | 1.4 | 1.1 | 1.6 | N/A | N/A |
| area 1 (day 1) | 6.2 | 4.9 | 4.0 | 3.9 | 3.4 |
| area 2 (day 1) | 1.7 | 2.9 | 5.9 | 5.5 | 5.6 |
| area 1 (day 2) | 10.6 | 5.4 | 4.5 | N/A | N/A |
| area 2 (day 2) | 9.1 | 4.6 | 2.9 | N/A | N/A |
| area/t1(day 1) | 5.6 | 4.7 | 2.9 | 3.7 | 3.4 |
| area/t2 (day 1) | 1.8 | 3.0 | 4.9 | 5.1 | 5.6 |
| area/t1(day 2) | 10.9 | 6.4 | 3.3 | N/A | N/A |
| area/t2 (day 2) | 9.3 | 5.4 | 2.3 | N/A | N/A |
| **Repeatability (interday, n=12)** |  |  |  |  |  |
| t1 | 1.9 | 1.6 | 1.7 | N/A | N/A |
| t2 | 1.9 | 1.6 | 1.8 | N/A | N/A |
| area 1 | 20.5 | 5.1 | 4.1 | N/A | N/A |
| area 2 | 18.0 | 3.9 | 4.4 | N/A | N/A |
| area/t1 | 22.0 | 6.0 | 3.0 | N/A | N/A |
| area/t2 | 19.6 | 4.2 | 4.9 | N/A | N/A |

^†^) data not available
